# Development of a Web-Based Intervention to Support Primary Health Care Professionals in Digital Health Measurement: User-Centered Participatory Approach

**DOI:** 10.2196/72331

**Published:** 2025-09-16

**Authors:** Kristel Meijers, Esther Bols, Emmylou Beekman, Rachel Slangen, Peter Alexander van de Hoef, Darcy Ummels, Rik Crutzen

**Affiliations:** 1 Research Center for Autonomy and Participation for Persons with a Chronic Illness Academy for Speech and Language Therapy Zuyd University of Applied Sciences Heerlen The Netherlands; 2 Department of Health Promotion Care and Public Health Research Institute Maastricht University Maastricht The Netherlands; 3 Academy for Physiotherapy Zuyd University of Applied Sciences Heerlen The Netherlands; 4 Department of Family Medicine Care and Public Health Research Institute Maastricht University Maastricht The Netherlands; 5 Research Center for Personalized Lifestyle and Movement-based Care Academy for Physiotherapy Zuyd University of Applied Sciences Heerlen The Netherlands; 6 Research Group Innovation of Human Movement Care Research Center for Healthy and Sustainable Living HU University of Applied Sciences Utrecht Utrecht The Netherlands; 7 Department of Rehabilitation Medicine Care and Public Health Research Institute Maastricht University Maastricht The Netherlands

**Keywords:** cocreation, digital health, eHealth, health measurement, general practitioners, implementation, language therapy, occupational therapy, participatory action research, physical therapy, primary health care, support, telemedicine, telemonitoring, user-centered design

## Abstract

**Background:**

Digital health measurement offers opportunities to address several primary health care challenges, but health care professionals encounter significant implementation barriers. Therefore, resources need to be developed to facilitate the integration of digital health measurement into daily practice.

**Objective:**

We aim to identify the most appropriate format and content for an intervention to support primary health care professionals in adopting digital health measurement. In addition, we describe and reflect on the development process.

**Methods:**

We used a participatory action research approach as well as user-centered design principles. A total of 19 primary health care professionals from 4 disciplines—physical therapy, occupational therapy, speech and language therapy, and general practitioner practice assistance—participated in intervention development as end users. External experts were consulted to broaden perspectives. Data were collected across 3 iterative stages (concept, design, and testing and trials) between January 2022 and December 2023 during cocreative meetings, individual interviews, focus group discussions, usability testing, and prototype use in daily practice. Data were analyzed using content analysis and descriptive statistics.

**Results:**

A web-based, stepwise intervention combining theoretical information, practical aids, examples, and experiences proved most suitable. Key features were concise content, intuitive and attractive graphic design, and flexible navigation and functionalities. Iterative improvements led to an increase in usability ratings from “okay” to “good to excellent.”

**Conclusions:**

Different health care disciplines benefit from similar support strategies; yet, this requires a careful balancing of intervention design and content. Combining participatory action research and user-centered design principles was useful to tailor the intervention to end users’ daily routines. The described development process offers a replicable framework for creating support strategies for digital health measurement in various health care settings.

## Introduction

### Background

Health care in the Netherlands faces multifaceted challenges that mirror global trends. These challenges include maintaining quality, accessibility, and affordability amid rising demand for care and the scarcity of health care professionals [1-3]. These system-wide pressures are particularly acute in primary care, partly due to the concurrent movement toward personalized health care provided in the home environment [2,4-6]. To address these challenges, national and international governmental bodies, alongside professional associations and research institutes, advocate for the implementation of digital health as a partial remedy [6-11].

Digital health encompasses the use of digital technologies for a diverse array of health-related functionalities, such as patient education, health status monitoring, and the management of patient records [7,12,13]. Preconditions for the effective use of digital health vary across functionalities and health care settings [7,11]. Consequently, this study focuses on one of these functionalities in a specific setting: the use of digital health for measurement purposes within primary health care.

In the Netherlands, primary health care comprises various health care disciplines and operates within a gatekeeping model, where general practitioners (GPs) coordinate care and manage referrals to hospital-based or specialist services. Primary care is largely delivered by independent, privately practicing GPs and other frontline professionals—such as physical therapists and occupational therapists—and is generally accessible to patients without prior GP referral.

Measurement outcomes guide the treatment process by providing diagnostic, prognostic, and evaluative information and by supporting shared decision-making [14,15]. However, to be effective, measurements must be goal oriented and meaningful [14,15]. This requires alignment with patients’ needs and objectives and an understandable presentation of measurement outcomes to health care professionals as well as patients and their significant others. Although assessing the effects of digital health use proves to be complicated [4,16], empirical evidence—such as studies on symptom monitoring demonstrating improvements in patients’ awareness, self-management, and treatment satisfaction in chronic conditions [6,17,18]—suggests objective benefits, while health care professionals themselves report perceived positive effects of digital health measurement on quality of care, efficiency, patients’ self-management, and prevention [3,11,19].

Despite generally positive attitudes of both health care professionals and patients toward digital health [3,11,19-22] and the widespread availability of digital health measurement instruments [6,7,23,24], the integration of these instruments into daily practice lags behind due to implementation barriers in various domains [6,11,21,22]. These barriers include technical, organizational, legal, and financial challenges as well as deficits in knowledge, skills, training, and time [6,20,25,26]. Furthermore, the sheer number of available digital health measurement instruments can itself pose an implementation barrier. The literature indicates that health care professionals, regardless of discipline or type of digital health technology, feel overwhelmed by the growing number of available instruments [16,24,27]. An informal survey among primary health care professionals in the Netherlands—comprising physical therapists, occupational therapists, speech and language therapists, and GP practice assistants—confirmed this sentiment for digital health measurement. Consequently, the clinical decision-making process, which involves goal-oriented identification, evaluation, and the selection of appropriate digital health measurements tailored to specific purposes and contexts, becomes challenging [27]. Hence, there is an urgent need to develop support strategies to address these barriers and promote the sustainable implementation and meaningful use of digital health measurement in daily practice [22,28,29].

### Objectives

While some resources are available to support primary health care professionals in the Netherlands with aspects of digital health use, no overarching intervention currently exists that (1) enables the implementation of digital health measurement across primary health care disciplines and regardless of use context, (2) integrates relevant strategies and resources to guide professionals in assessing whether and how to use digital health measurement to inform and enhance patient management in real-world practice, and (3) is freely accessible to all intended users. Furthermore, the appropriate format and content for such an intervention—tailored to seamlessly integrate into daily clinical routines and address key barriers faced by primary health care professionals—remain undefined. Therefore, we aim to determine the optimal format and content for an intervention to support sustainable and goal-oriented digital health measurement in primary practice and to describe and reflect on the intervention’s development process. The study is reported in accordance with the SRQR (Standards for Reporting Qualitative Research) checklist [30].

## Methods

### Design

To align with the needs of primary health care professionals, the intervention was developed using a bottom-up approach rooted in participatory action research (PAR) [31]. PAR entails equitable collaboration between researchers and stakeholders [32]. It involves iterative, overlapping cycles of planning and implementing change, followed by evaluation, reflection, and adjustment. PAR aims to both generate knowledge and drive meaningful practice change [31,33-36]. We used PAR to identify preferred strategies and resources for supporting the implementation of digital health measurement while enhancing its sustainable and goal-oriented use in daily practice. In this study, digital health measurement refers to (1) the use of digital questionnaires, electronic patient-reported outcome measures, measurement tools, apps, wearables, and websites in practice settings or at patients’ homes (2) by primary health care professionals alone or with their patients (3) to measure health conditions, body functions, body structures, activities, participation, and environmental or personal factors [37] (4) for diagnostic, treatment, and evaluation purposes [12,38].

To ensure that the intervention is user-friendly and tailored to real-world contexts, user-centered design (UCD) principles were incorporated into the PAR approach [39,40]. UCD emphasizes considering implementation conditions from the outset and testing easily understandable prototypes in their intended context. User involvement spans 4 UCD stages: concept, design, testing and trials, and deployment [39,41]. This paper focuses on the first 3 stages, as they form the foundation of the intervention’s development. Deployment will be addressed in a subsequent evaluation.

The study is part of a broader PAR initiative that also investigates the intervention’s implementation, effectiveness, and impact in real-world primary care settings. Within this broader framework, end users were involved actively not only in designing the intervention but also in co-defining the research problem, aims, and questions as well as in participating in the study’s evaluation. Their level of participation encompassed advisory input (eg, sharing adjustment proposals), partnership (eg, codeciding on intervention format), and control (eg, defining relevant support needs and content) [42,43].

### Ethical Considerations

All procedures involving human participants were reviewed and approved by the local medical research ethics committee, Medisch Ethische Toetsings Commissie Zuyderland (METCZ20220049 and METCZ20220112). All participants provided written informed consent after receiving detailed written information about the study (in Dutch), including their right to withdraw at any time without consequence. All data were pseudonymized by replacing identifiable information—such as the names and locations of health care professionals and practices—with unique codes in both the transcripts and contextual datasets. Participants received partial financial compensation only for the time invested in study activities that extended beyond their regular professional responsibilities. No images or other media containing identifiable individual participants are included in this manuscript or supplementary materials.

### Participants

#### PAR Team

Typically, in PAR, researchers and those participating in the research cooperate as a team [32,33,35]. In this study, the initial PAR team consisted of primary health care professionals, participating as end users, and researchers from Zuyd University of Applied Sciences, Utrecht University of Applied Sciences, and Maastricht University. All researchers brought extensive and diverse expertise relevant to the study. As the intervention evolved, additional specialists, including a communication expert and website developers, joined the team to meet emerging needs.

#### Recruitment of End Users

To promote the applicability of the intervention across Dutch primary health care, recruitment aimed to ensure diversity in both health care disciplines and the characteristics of practices and end users. Three quartets were to be formed, each consisting of 1 practice from 4 distinct health care disciplines: physical therapy, occupational therapy, speech and language therapy, and GP practice assistance. These disciplines were selected because they commonly use patient measurements to guide treatment, have comparable roles and responsibilities in delivering individualized care, and collaborate interprofessionally within Dutch primary care. Collectively, they represent a subset of disciplines with the highest volume of care delivery, the most frequent patient contact, and some of the fastest professional growth within the Dutch primary care system [44]. While their roles are comparable and span both prevention and treatment, their clinical foci differ. GP practice assistants—a discipline introduced in the Netherlands in 1999 and less commonly known internationally—independently perform basic clinical tasks previously carried out by GPs, referring patients back to the GP when more complex care is required [45,46]. With 77% to 80% of their time dedicated to patient-specific activities, their role is primarily clinical. Practices were eligible for inclusion if they were located near the main research facility in the Dutch province of Limburg and willing to actively engage in intervention development and testing. Quartets were to be enrolled sequentially, allowing each to build on and validate insights from earlier enrollees.

Before the study, quartets 1 and 2 were purposively recruited [47] through the researchers’ professional networks based on health care discipline and variation in practice and professional characteristics. During the study, quartet 3 was also purposively recruited through the networks of both researchers and end users. Practices were assigned to quartets based on discipline and availability. The practices independently determined which and how many of their health care professionals would participate.

#### External Experts

An advisory board provided feedback throughout all stages of intervention development. The board consisted of representatives from key national professional associations [48-51], a human movement scientist, and 2 professors specialized in digital health care innovations and the implementation of clinical guidelines in general practice. In addition, human factors engineers, lecturers, students, researchers, and primary health care professionals from Zuyd University of Applied Sciences, Utrecht University of Applied Sciences, Windesheim University of Applied Sciences, and Maastricht University provided feedback based on their areas of expertise at various points during the development process. This collaborative effort aimed to broaden perspectives and address end users’ time constraints.

### Data Collection and Analysis

#### Overview

Data were collected and analyzed between January 2022 and December 2023 during multiple sessions at various sites in the Netherlands. The following participant characteristics were documented: end users’ sex, age, work experience, and technology user personas [52] as well as practices’ size (number of staff and locations), socioeconomic status (SES) score, and expertise.

Throughout all UCD stages, participants’ perspectives on the planned intervention were collected and analyzed using various approaches tailored to the specific objectives of each stage, the type of data required, the depth of analysis warranted, the emerging group dynamics, and time constraints; for instance, during the concept and design stages, sessions were intentionally loosely structured, with data primarily collected through forms, minutes, and photographs. This approach aimed to foster creativity, minimize inhibition among participants, and support an egalitarian researcher-participant relationship. During the testing and trials stage, data collection focused on testing instead of cocreating and thus primarily relied on verbatim transcripts to allow for more rigorous analysis. In a few cases, the need to ensure inclusive input and accommodate participant availability led to the unanticipated addition of individual sessions.

Data analysis was performed through deductive-inductive content analysis, descriptive statistics, or predefined criteria. The initial deductive coding drew on theoretical models, commonly used categories in relevant literature, team expertise, and study priorities to define broad, non–topic-specific categories, leading to categories such as skeleton plane [53,54], barriers, facilitators, support questions, and professional journey. Inductive coding was then used to refine or expand these with content-specific categories or subcategories based on participant input—such as data presentation, website title, and user-input features—ensuring both theoretical grounding and contextual relevance. The aim was to collect and analyze data until saturation was reached. Saturation was determined by reflexive discussions among the researchers during data collection and analysis, based on data saturation (repetition of data) as well as meaning saturation (adequacy and completeness of themes as well as their meaning and coherence with other themes) [55,56]. Detailed descriptions of data collection and analysis per stage are provided in the following subsections.

#### Concept Stage

The concept stage aimed to generate an initial outline for intervention development. Two iterations were conducted (Table 1). In the first iteration, the “six thinking hats” method [57-59] was used by end users and the advisory board during a cocreative session to share experiences on digital health measurement as well as intervention format and content needs. This method invites participants to adopt different perspectives (eg, benefits, caution, and creativity)—symbolized by 6 colored hats—to encourage structured and multifaceted input. Participants were prompted to metaphorically switch hats to reflect on the topics from different angles. This session was augmented by individual conversations to deepen insights and ensure input from all end users.

**Table 1:** Aims, methods, data collection methods, participants, and analysis approaches for each iteration across the concept, design, and testing and trials stages.

Stages and iterations and aims				Methods		Data collection methods		Participants		Analysis
**Concept stage**										
	**Iteration 1**									
		Gaining insight into digital health measurement experiences and intervention format and content needs	“Six thinking hats” [57,59] method, sensitizing preparatory questions		Minutes and forms		End users (quartet 1) and advisory board		Deductive-inductive content analysis [60]	
		Gaining insight into digital health measurement experiences and intervention format and content needs	Semistructured individual conversations		Minutes		End users (quartet 1)		Deductive-inductive content analysis [60]	
	**Iteration 2**									
		Refining intervention content needs to create low-fidelity prototype 1	MoSCoW^a^ method [59,61], brainstorm session, and plenary discussion		Photographs, forms, and minutes		End users (quartet 1)		Predefined MoSCoW criteria	
		Refining intervention format needs to create low-fidelity prototype 1	Plenary discussion		Minutes		End users (quartet 1)		Deductive-inductive content analysis [60]	
		Refining intervention content needs to create low-fidelity prototype 1	MoSCoW method and plenary discussion		Minutes		Advisory board		Predefined MoSCoW criteria	
		Refining intervention format needs to create low-fidelity prototype 1	Plenary discussion		Minutes		Advisory board		Deductive-inductive content analysis [60]	
**Design stage**										
	**Iteration 1**									
		Enhancing visual aesthetics, structure, and content of medium-fidelity prototype 1	Independent website visit, content review^b^, and semistructured individual conversations		Reviewed documents and minutes		End users (quartet 1)		Deductive-inductive content analysis [60]	
		Enhancing visual aesthetics, structure, and content of medium-fidelity prototype 1	(1) Independent website visit and plenary discussion or, based on availability of participants, (2) independent website visit, sensitizing preparatory questions, and semistructured individual conversations		(1) Minutes or (2) forms and minutes		Advisory board		Deductive-inductive content analysis [60]	
		Enhancing visual aesthetics, structure, and content of medium-fidelity prototype 1	Website demonstration and plenary discussion		Minutes		End users (quartet 2)		Deductive-inductive content analysis [60]	
	**Iteration 2**									
		Enhancing visual aesthetics, structure, and content of medium-fidelity prototype 2	Codiscovery^c^ [62,63], SUS^d^ [64], and plenary discussion		Forms, questionnaires, and minutes		Session 1: external experts; session 2: end users (quartets 1 and 2); session 3: external experts		Forms and minutes: inductive content analysis [60]; SUS: descriptive statistics	
		Enhancing visual aesthetics, structure, and content of medium-fidelity prototype 2	Codiscovery and plenary discussion		Forms, logs and minutes		Advisory board and external experts		Inductive content analysis [60]	
		Enhancing visual aesthetics, structure, and content of medium-fidelity prototype 2	Plenary discussion		Minutes		External experts		Inductive content analysis [60]	
		Enhancing content of medium-fidelity prototype 2	Content review^e^		Reviewed documents		End users (quartet 1)		Inductive content analysis [60]	
**Testing and trials stage**										
	**Iteration 1**									
		Optimizing usability and persuasive design principles of high-fidelity prototype 1	Heuristic evaluation		Minutes and quotes		External experts		Summary for each heuristic, illustrated by quotes, and matched with recommendations for improvement	
		Optimizing surface and skeleton of high-fidelity prototype 1 [53]	Individual usability testing and SUS		Verbatim transcripts of audio recordings and questionnaires		End users (quartets 1 and 2) and external experts		Verbatim transcripts: deductive-inductive content analysis [60]; SUS: descriptive statistics	
		Optimizing content of high-fidelity prototype 1	Website^f^ use in daily practice supported by coaching on the job and focus group discussion		Minutes (coaching on the job) and verbatim transcripts of audio recordings (focus group discussion)		End users (quartets 1, 2, and 3)		Directed content analysis [65]	
	**Iteration 2**									
		Optimizing structure and scope of high-fidelity prototype 2 [53]	Individual usability testing and SUS		Verbatim transcripts of audio recordings and questionnaires		End users (quartets 1, 2, and 3)		Verbatim transcripts: deductive-inductive content analysis [60]; SUS: descriptive statistics	
		Optimizing content of high-fidelity prototype 2	Independent prototype use and focus group discussion		Verbatim transcripts of audio recordings		End users (quartets 1, 2, and 3)		Directed content analysis [65]	

^a^MoSCoW: must have, should have, could have, would have.

^b^Content pages in Microsoft Word or Microsoft Excel format, part 1.

^c^During the codiscovery sessions, pairs of participants were asked to write down their first impressions, actions, and experiences while first navigating freely through the website and then solving a practice-based measurement case using the website.

^d^SUS: System Usability Scale.

^e^Content pages in Microsoft Word or Microsoft Excel format, part 2.

^f^Quartet 1 used medium-fidelity prototype 3, quartet 2 used medium-fidelity prototype 3 and high-fidelity prototype 1, and quartet 3 used high-fidelity prototype 1.

In the second iteration, group meetings were conducted to refine content and format needs. The MoSCoW (must have, should have, could have, would have) method [58,59,61] was used by end users and the advisory board to prioritize content needs. Needs rated as “must have” by at least 1 participant or “should have” by at least 2 participants were considered crucial for the intervention. Subsequently, end users were asked to identify content categories relevant to their needs during brainstorming sessions, followed by a plenary discussion. Finally, end users and advisory board members were invited to deliberate on further preferences for the intervention format. As an outline for intervention development, content needs and matching content categories were organized in low-fidelity prototype 1.

To support end users to take implementation conditions into account during the design stage, the researchers also created the initial version of a professional journey [59] that linked intervention use to clinical practice.

#### Design Stage

To foster conceptual understanding and consensus on the intervention’s scope and content while following UCD principles, the researchers developed realistic medium-fidelity prototypes early in the design stage. As end users preferred a web-based intervention format, a communication specialist was integrated into the PAR team to support prototype development. From this stage onward, all prototypes consisted of a website with a structured plan for digital health measurement at its core, accompanied by content pages and progressively added functionalities. Each prototype underwent initial evaluation and refinement by the researchers before presentation to end users or external experts.

In 2 iterations (Table 1), the design stage centered on enhancing the visual aesthetics, structure, and content of 2 subsequent medium-fidelity prototypes to fit the intervention’s objectives. In the first iteration, end users and members of the advisory board participated in individual conversations (to accommodate availability), plenary discussions, and content reviews to comment on medium-fidelity prototype 1. Furthermore, the initial professional journey was reviewed, and revised into its final version (Multimedia Appendix 1). In the second iteration, medium-fidelity prototype 2 was assessed by end users, advisory board members, and external experts at the 3 collaborating universities of applied sciences using codiscovery [62,63], plenary discussion, and content reviews. In 3 sessions, participants also completed the System Usability Scale (SUS) [64], which had been translated into Dutch for this purpose. These quantitative data were collected to support qualitative data, and both provided input for a final plenary discussion debating overall experiences.

Two researchers analyzed data from each iteration and deliberated to formulate initial proposals for prototype adjustment based on the analyzed data. These findings were member checked and then discussed among all researchers and the communication specialist until consensus was reached on the changes to implement in the next prototype. Agreed-upon changes were then, where possible, integrated into medium-fidelity prototype 3; for example, when analysis revealed diverging preferences regarding the level of content detail in medium-fidelity prototype 2, the researchers and communication specialist reviewed the corresponding adjustment proposals. Suggestions that were considered to reconcile these differences were selected by consensus and incorporated into the subsequent prototype for evaluation in the next iteration.

#### Testing and Trials Stage

##### Overview

In light of the developmental stage and the complexity of some of the required alterations, professional website developers were added to the PAR team. They incorporated all required modifications from the design stage into the initial high-fidelity version of the website. Simultaneously, testing commenced using medium-fidelity prototype 3 to allow for a timely progression of the testing and trials stage.

The testing and trials stage involved 2 iterations (Table 1) primarily aimed at refining the prototype’s visual aesthetics, structure, and content and functionalities for practical use and eventual dissemination. This stage included heuristic evaluation, usability testing, and website use in daily practice. Throughout this stage, minor adjustments were continually implemented to the prototype layout.

##### Heuristic Evaluation

Heuristic evaluation sought to identify potential issues related to both usability and persuasive design principles. Lecturers and second-year students in communication and multimedia design scrutinized the navigation structure, forms, layout, and use of visual elements in high-fidelity prototype 1. They used the 10 usability heuristics formulated by Nielsen [66] as well as the 6 persuasive heuristics formulated by Cialdini [67] to assess the prototype (Multimedia Appendix 2 [66,67]). Subsequently, they correlated the usability and design problems that had been identified with recommendations for improvement.

##### Usability Testing

The framework for website design developed by Garrett [53] and the study by Crutzen et al [54] were used as guides for usability testing. The framework consists of 5 planes, broadly referring to the following elements: from the bottom up, the strategy plane pertains to the website’s objectives and the needs it addresses. This informs the scope plane, which defines the content and functionalities to be incorporated. The structure plane then determines how these elements are linked to work together. The skeleton plane translates this structure into layout by placing elements at specific locations and enabling end users to navigate the website. Finally, the surface plane pertains to the website’s visual appearance. User experiences occur at each of these planes but are typically perceived from the top down, starting with the surface plane.

Usability testing involved individual sessions with end users as well as lecturers and researchers. Each session consisted of free navigation through the website, problem-solving while synchronously thinking aloud [59], completion of the translated SUS [64], and an interview. During the first iteration, the interview covered the surface and skeleton planes of the framework developed by Garrett [53]; during the second iteration, it focused on the structure and scope planes.

##### Website Use in Daily Practice

In the first iteration, end users used either medium-fidelity prototype 3 or high-fidelity prototype 1 in their daily practice to tackle self-selected digital health measurement challenges. Simultaneously, they received coaching-on-the-job support from 2 researchers. In this early stage of intervention development [68], coaching on the job was applied as a temporary implementation strategy to ensure the timely progression of prototype testing and facilitate comprehensive data collection on development issues. The 3 quartets of end users initiated prototype testing sequentially, with each quartet commencing 2 months apart. During the coaching-on-the-job sessions and subsequent focus group discussions per quartet, data were collected on website use experiences, focusing on the content requirements delineated in the scope plane of the framework developed by Garrett [53].

In the second iteration, all end users used high-fidelity prototype 2 independently for 2 months to address any remaining or new digital health measurement challenges within their daily practice. Their experiences were discussed during 2 focus group discussions in which all quartets were mixed.

On the basis of the analyzed results from each iteration and evaluation, the researchers and website developers discussed which prototype adjustments would be suitable and feasible within the study context. These adjustments were incorporated into high-fidelity prototype 2 (adjustments from iteration 1) and the final website version (adjustments from iteration 2).

### Trustworthiness

Efforts were made to safeguard the credibility, confirmability, transferability, and dependability of the research [69-71]. Researchers and end users worked together on a regular basis during a 2-year period. Several researchers had prior professional or academic experience in the health care disciplines under study and had investigated the implementation of health measurement in primary care over multiple years. This insider perspective enriched the understanding of the context in which the intervention was to be used. Credibility was also bolstered by using various data sources, methods of data collection, and investigator triangulation. All data were collected, analyzed, and interpreted by a minimum of 2 investigators. Throughout the research process, findings were continually shared with end users and external experts for validation through real-time summaries and member checking of minutes and transcripts [69]. In addition, interpretations and conclusions derived from the data underwent member checking in follow-up sessions that involved oral presentations and the presentation of subsequent prototypes. Peer debriefing and reflexive notes, for instance on insider and outsider positionality, were used to augment confirmability. To mitigate potential limitations associated with positionality, researchers with more outsider perspectives—unfamiliar with the specific health care disciplines or settings—were intentionally included. This diversity also extended to differing levels of experience with and attitudes toward digital health among all PAR team members, ensuring a broader range of insights throughout the study. To facilitate transferability, a “rich description” of the characteristics of the end users and their practices was provided. To ensure the dependability of the research findings, an emergent research design was adopted, with decisions on the next steps based on earlier results. The sequential inclusion of quartets allowed for iterative data collection and analysis.

## Results

### Participant Characteristics, Data Collection, and Data Analysis

A total of 19 health care professionals across 11 practices were recruited as end users (Tables 2 and 3). These participants engaged their colleagues by sharing experiences with intervention use, encouraging them to use the intervention, and incorporating peer feedback into its further development. In total, 89 health care professionals were involved through this process of peer engagement. Quartets 1 and 2 each included 4 practices representing all 4 health care disciplines under examination. Quartet 3 incorporated 3 practices and health care disciplines. Despite extensive recruitment efforts, a GP practice assistant could not be enlisted in quartet 3. The reasons given for inability to participate included workload constraints and participation in concurrent research projects. Table 2 demonstrates that the recruited end users displayed a variety of ages, work experiences, and experiences and attitudes toward digital health. In addition, the sample represented a range of practice sizes, expertise, and SES scores based on the practice’s postcode areas (Table 3). Two end users (the speech and language therapist in quartet 1 and the GP practice assistant in quartet 2) withdrew from the study—one at the beginning of the testing and trials stage and one midway through the testing and trials stage—due to organizational challenges (significant staff turnover) and health-related reasons. These individuals represented 2 of the 11 participating practices (18%) and 2 of the 19 end users (11%).

**Table 2:** Demographic, occupational, and technology-related characteristics of the end users (N=19).

Quartets^a^ and end users (technology user persona [52])		Sex	Age (y)	Work experience in health care discipline (y)
**Quartet^b^ 1**				
	PT^c^1.1 (digitally proficient professional)	Male	66	43
	PT1.2 (digitally proficient professional)	Male	28	9
	PT1.3 (digitally proficient professional)	Female	24	5
	PT1.4 (digitally proficient professional)	Female	38	17
	PT1.5 (hesitant technology user)	Female	31	10
	OT^d^1 (digital enthusiast)	Female	43	20
	SLT^e^1 (digital enthusiast)	Male	63	9^f^
	GP^g^ practice assistant 1 (digitally proficient professional)	Female	51	16
**Quartet 2^h^**				
	PT2.1 (digital enthusiast)	Female	24	2
	PT2.2 (hesitant technology user)	Female	37	16
	OT2 (digital enthusiast)	Female	36	14
	SLT2 (hesitant technology user; digitally proficient professional)	Female	54	30
	GP practice assistant 2 (digitally proficient professional)	Female	64	16
**Quartet 3^i^**				
	PT3.1 (digitally proficient professional; digital enthusiast)	Male	29	7
	PT3.2 (hesitant technology user)	Female	45	25
	OT3.1 (digitally proficient professional)	Male	26	5
	OT3.2 (digitally proficient professional)	Male	27	5
	SLT3.1 (digital enthusiast)	Female	42	21
	SLT3.2 (digitally proficient professional)	Female	49	26

^a^The term *quartet* refers to the number of primary health care practices and health care disciplines that were intended to be included in each group. It does not reflect the number of end users in each group.

^b^Quartet 1’s median age and work experience in health care discipline (years) are 40.5 (range 24-66) and 13 (range 5-43), respectively.

^c^PT: physical therapist.

^d^OT: occupational therapist.

^e^SLT: speech and language therapist.

^f^SLT1’s work experience refers to their role as a practice manager in an SLT practice.

^g^GP: general practitioner.

^h^Quartet 2’s median age and work experience in health care discipline (years) are 37 (range 24-64) and 16 (range 2-30), respectively.

^i^Quartet 3’s median age and work experience in health care discipline (years) are 35.5 (range 26-49) and 14 (range 5-26), respectively.

**Table 3:** Organizational and contextual characteristics of the participating primary health care practices (N=11).

Quartets and practices			Health care professionals employed, n		Practice locations, n		SES^a^ scores^b^		Expertise
**Quartet 1**									
	Physical therapy	Physical therapists: 10		5		Range^c^ −0.543 to 0.209		Pelvic health, manual therapy, pediatrics, geriatrics, neurology, COVID-19, edema, medical taping, intermittent claudication, preoperative care, rehabilitation, vocational rehabilitation, and lifestyle intervention	
	Occupational therapy	Occupational therapists: 3		2		0.122 and 0.135		Workplace ergonomics, pediatrics, neurology, compression stockings, mental health, chronic pain, and COVID-19	
	Speech and language therapy	Speech and language therapists: 7		20		Range −0.625 to 0.135		Voice, speech, language, swallowing, hearing, orofacial myology, dyslexia, and dyscalculia	
	General practitioner practice assistance	General practitioners: 3; general practitioner in training: 1; general practitioner’s assistants: 6; and general practitioner practice assistants: 5		1		0.028		General care and chronic care (diabetes mellitus type 2, cardiovascular diseases, geriatrics, mental health, and pulmonary conditions)	
**Quartet 2**									
	Physical therapy	Physical therapists: 8; occupational therapists: 3; speech and language therapists: 4; osteopath: 1; and exercise therapist: 1		4		Range −0.439 to 0.215		Oncology, pulmonary conditions, neurology, Parkinson disease, chronic pain, edema, orthopedics, headache, hand therapy, intermittent claudication, and holistic coaching	
	Occupational therapy	Occupational therapists: 12		1		−0.248		Sensory processing, dementia, neurology, pediatrics, osteoarthritis, and rheumatism	
	Speech and language therapy	Speech and language therapist: 1		1		−0.469		Speech, language, and dyslexia	
	General practitioner practice assistance	General practitioners: 3; general practitioner’s assistants: 4; and general practitioner practice assistants: 3		1		0.061		General care and chronic care (diabetes mellitus type 2, cardiovascular diseases, geriatrics, mental health, and pulmonary conditions)	
**Quartet 3**									
	Physical therapy	Physical therapists: 7		1		−0.310		Geriatrics, oncology, edema, hand therapy, pulmonary conditions, manual therapy, pelvic health, headache, (chronic) pain, orthopedics, osteoarthritis, neurology, cardiovascular diseases, postoperative care, and lifestyle intervention	
	Occupational therapy	Occupational therapists: 2		2		−0.258 and 0.036		Mental health, occupational health and prevention, pediatrics, oncology, neurology, dementia, COVID-19, osteoarthritis, and rheumatism	
	Speech and language therapy	Speech and language therapists: 5		3		Range −0.354 to 0.045		Voice, speech, language, breathing, swallowing, globus pharyngeus, neurology, facial palsy, and dyslexia	

^a^SES: socioeconomic status.

^b^The average scores for the practice locations’ postcode areas [72] are shown. The score for a postcode area is the average of all household scores within that area. The average SES-WOA (Wijk- en Omgevingsanalyse; Dutch for Neighborhood and Environmental Analysis) score for the Netherlands is 0. Scores typically range from −2 to 1. Higher scores indicate higher wealth, education, and employment duration.

^c^The range represents the lowest and highest average SES scores of the practice’s locations if it has >2 locations.

The duration of data collection sessions varied: 30 to 75 minutes for usability testing and individual interviews, 30 to 120 minutes for group discussions, 60 to 120 minutes for cocreative sessions and codiscovery, and 240 minutes for heuristic evaluation. Data were collected and analyzed from all participants until responses no longer provided new insights related to the stage’s objectives. At the end of each stage, categories resulting from data analysis reflected general agreement on which aspects of the prototype needed improvement and why, although, in a few instances, there was no consensus on how to execute these improvements. Nonetheless, depth of understanding was deemed sufficient to progress with iterative prototype development [55,56]. Iteration continued until only minor issues were reported, and usability scores were “good to excellent.” Multimedia Appendices 3-5 visualize website development by providing illustrative screenshots of key components from 2 iterative prototypes as well as the final website version.

### Concept Stage

The data collected through the “six thinking hats” method [57-59] and individual conversations in the first iteration provided insight into barriers, facilitators, content and format needs, and good practices regarding the use of digital health for measurement purposes.

Table 4 summarizes the barriers and facilitators identified as relevant to our bottom-up approach to enhancing digital health measurement. Throughout all developmental stages, prototype format and content were tailored to mitigate these barriers and leverage these facilitators. For instance, adjustments were made to accommodate time constraints at the practice level, ensuring the intervention’s efficiency. Furthermore, the intervention content was customized to promote goal-oriented use of digital health measurement, which the end users considered a facilitator for digital health measurement.

**Table 4:** Barriers and facilitators to digital health measurement in primary care, as identified by end users and advisory board members.

Barriers			Facilitators
**Health care professional**			
	Lack of knowledge and skills concerning several topics (general unfamiliarity; lack of experience; digital skills; patient selection; finding and selecting measurement instruments; goal-oriented use; recording, selecting, and presenting measurement data; and implementation) and attitude	Innovation and oversight generate positive feelings; more targeted coaching and therapy	
**Patient**			
	General unfamiliarity; applicability for target audience; attitude; and readiness for digital health measurement (understanding of what is expected)	Added value; quality of care; registration and presentation of measurement data; wide applicability (goals and target audiences); more goal-oriented therapy; and positive experiences	
**Practice**			
	Time constraints and excessive transitions in primary care	Efficiency; reputational benefit; and consistency with digital skills within team	
**Characteristics of digital health measurement instruments**			
	Supply and clinimetric quality	Possible uses; quality of measurements; functionalities for registration, analysis, communication, and feedback; and efficiency	
**Policy and outer context**			
	Legal aspects (privacy and General Data Protection Regulations); little scientific evidence; and lack of resources providing oversight	Consistency with policies of external parties	

Content needs shared by end users and members of the advisory board were translated into 23 support questions (Multimedia Appendix 6). Concerning intervention format, end users expressed the need for an interdisciplinary stepwise plan, flowchart, or checklist. These content and format needs were elaborated on during the second iteration of the concept stage and guided prototype development during the design stage. The identified good practices encompassed various aspects of digital health use, namely examples of digital health measurement instruments, implementation processes, benefits for patients, listing sites, and financing options. These good practices were used and expanded on during the testing and trials stage to inspire intervention application in daily practice.

In the second iteration, MoSCoW ratings [59,61] indicated that all 23 support questions were essential for the intervention (Multimedia Appendix 6). The “10-step plan for integrated use of measurement instruments in practice” [14,15] was used to organize these support questions, as the plan aligned closely with both the content and format needs from the first iteration. Together, end users and researchers identified content categories to address the support questions. This process yielded the low-fidelity prototype of the intervention, which consists of a matrix that organizes the support questions according to the 10-step measurement plan and aligns them with content categories (Multimedia Appendix 6).

End users deliberated on diverse formats for the intervention. Ultimately, they agreed on a web-based intervention:

You need to choose a format for the intervention that has appeal: a website or app to generate enthusiasm among employees. It should be systematic and scientific, but wrapped in an attractive package.

Physical therapist, quartet 1

A hard copy I will put on a shelf, and I won’t look at it again, a clickable PDF I will save once and won’t come back to, a website I can access anytime.

Occupational therapist, quartet 1

### Design Stage

#### Overview

The first medium-fidelity prototype consisted of (1) a website home page with a matrix representing the 10-step plan for digital health measurement, linking to layers of basic and in-depth content pages for steps 1 and 2 as well as background information; and (2) Microsoft Word and Microsoft Excel documents containing proposed content for all steps. In the second medium-fidelity prototype, the content of the Word and Excel documents was added to the website’s basic or in-depth content layers. The prototypes integrated various types of content, including text, icons, pictures, and hyperlinks to authoritative websites and resources.

The data collected from end users and external experts during the design stage primarily focused on the visual aesthetics, structure, and content of medium-fidelity prototypes 1 and 2. Given the conceptual similarity of the reviewed prototypes across both iterations and the consistent participant feedback on the same topics, the qualitative results from iterations 1 and 2 are clustered per topic. Quantitative data were collected only for medium-fidelity prototype 2 during codiscovery sessions 1 to 3, using the SUS. Of the 14 participants of these sessions, 12 (86%) completed the SUS, resulting in a mean score of 58.3 (SD 11.4), which corresponds to an “okay” usability rating (percentile range 15-34) [73,74].

#### Visual Aesthetics

Participants widely regarded the overall aesthetics of the website as attractive, clear, and inviting. However, certain icons and images were considered incongruent with digital health and the primary health care context:

The first moving image is not illustrative, but it is attractive. The health care professional in that image is not using digital health, but papers. Something along these lines is nice.

Physical therapist, quartet 1

The use of bold font to signify key words led to confusion among participants.

#### Structure

Participants found the website structure clear, effective, and pleasant. They often used the website intuitively, without resorting to the provided instructions. However, they suggested making the stepwise plan more prominent because of its central role in the website’s goals and functioning:

Personally, I would like the 10 steps to be more explicitly visible without having to scroll. Now, attention is primarily directed to the title and a dynamic picture.

Professional association member

In addition, the flexibility for end users to either follow all steps or select specific ones was not immediately apparent. Furthermore, participants indicated a need for functionalities that allow experienced users to bypass steps and quickly locate relevant resources.

While participants appreciated the use of layers to differentiate between basic and in-depth information, they experienced disorientation within the website’s structure:

Lots of opportunities everywhere to continue clicking through the site, with examples and explanations, but you sometimes get lost as a user of the site.

Researcher

Participants requested improvements to the text structure as well as for quicker retrieval of relevant information, including revisiting the classification of basic and in-depth content. They also critiqued the use of full URLs for hyperlinks, suggesting keywords, tiles, or explanations for clarity.

#### Content

Overall, participants considered the website content relevant, comprehensive, and educational and appreciated the usefulness of the resources and their integration within the intervention. However, they suggested improvements to the website title and background information to better align with the intervention’s objectives and more clearly delineate digital health measurement.

End users reported that the stepwise plan addressed their support questions from the concept stage. While they found all steps useful, they identified additional relevant information and resources to include. Personal preferences varied regarding the balance of visual and textual information, detail level, and applicability to specific disciplines. Overall, participants felt that the content was too theoretical and necessitated translation into concrete, practical use:

The website now comes across as an educational tool to understand where to find what, what digital health is, and what is involved. It’s more of a study than a practical real-world tool.

Speech and language therapist, quartet 2

All very well reasoned, though maybe a little too well. Above all, you want to get started on your own question and it takes a long time to get there.

Researcher

Participants recommended using more accessible language, accompanied by practical, interdisciplinary examples. Moreover, they observed an overemphasis on generic measuring concepts, rather than highlighting focus areas for digital health measurement.

In terms of implementation conditions, participants raised concerns about the volume of information being incompatible with end users’ time constraints. Finally, they recommended minimizing links to paid resources to enhance user-friendliness.

**Figure 1:**
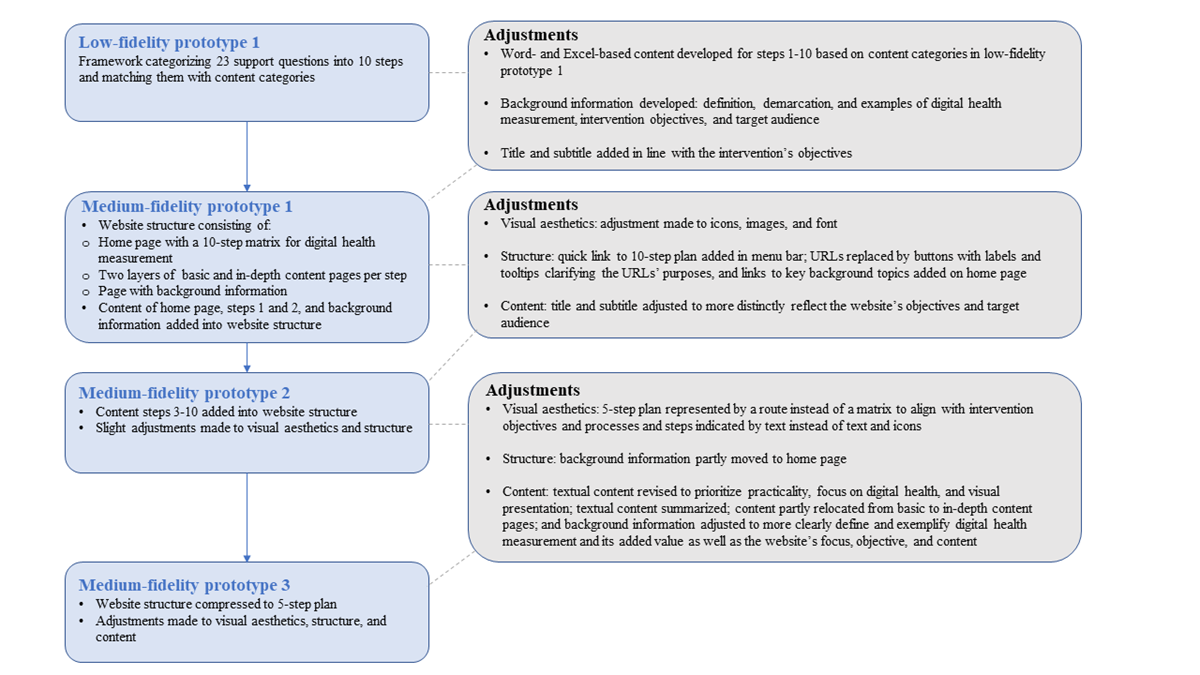
Adjustments made to iteratively advance the low-fidelity prototype of the intervention designed to support primary health care professionals in digital health measurement into medium-fidelity prototypes.

Integrating all findings, the prototype adjustments aimed to clarify the website’s goals and target audiences. They also aimed to balance content depth with user-friendly structuring of information to accommodate flexible and efficient use within the identified implementation conditions. Visual aesthetics, structure, and content of the subsequent medium-fidelity prototypes were revised, as illustrated in Figure 1. The final medium-fidelity prototype streamlined the website into a 5-step plan with overarching themes: formulating a measurement challenge, finding an appropriate digital health measurement instrument, assessing its quality, using the instrument and using its outcomes for shared decision-making, and implementing the instrument. Each step contained limited and practical content. Other requested adjustments were postponed for further exploration and integration during the testing and trials stage.

### Testing and Trials Stage

In the first iteration, the usability testing of high-fidelity prototype 1 yielded a mean SUS score of 76.6 (SD 5.8; 8/8, 100%), which indicates a “good” rating (percentile range 70-79) [73,74]. Qualitative data pertained to the categories of graphic design, navigation, and content and functionalities.

#### Visual Aesthetics

During the transition from medium-fidelity prototype 3 to high-fidelity prototype 1, professional website developers refined the graphic design. Participants in heuristic evaluation and usability testing praised the readability and ease of finding information, attributing this to user interface elements such as tooltips, colors, and headings. Tooltips and contrasting colors effectively highlighted supplementary information, thereby enhancing usability. However, participants noted inconsistencies in design across website layers, which disrupted the sense of calm and clarity, and they found it unclear at times whether graphic elements were intended for navigation or information purposes:

The design is calm, and the imagery reinforces the text and fits the context.

Human factors engineer 1

Imagery of the home page is calm and functional, but not thereafter.

Human factors engineer 2

While participants agreed that images should add value and reflect the website’s content and target audience, opinions diverged on which images fulfilled these criteria.

#### Structure

Heuristic evaluation and usability testing revealed that improving the website’s navigation structure—by enabling end users to track progress within the 5-step plan and offering multiple pathways to access information—would significantly enhance usability:

And here it would be convenient if I could already click through to the next one here. Because here I have to go back first, right? And then I have to go all the way back again. So, if clicking through from step one to step two could actually be on the same page would also be nice.

Lecturer

Participants suggested adding a menu bar to establish a standardized website structure and prominently feature the 5-step plan, emphasizing the website’s objectives. In addition, half of the participants considered the inclusion of a search function crucial for improving user-friendliness, usability, and efficiency.

#### Content and Functionalities

The potential to use social proof and authority in persuasive design was initially overlooked. Human factors engineers recommended integrating end users’ personal stories to enhance engagement and emphasizing the involvement of expert health care practices and research institutions in the intervention’s development to build trust:

Share reviews from others who have used this [intervention] and experienced in practice that they got better at using it [digital health measurement] or that it [digital health measurement] is clearer to them now.

Human factors engineer 1

There is no connection displayed to an organization that offers trust...Is there a link to that kind of party?

Human factors engineer 2

Usability testing and use in daily practice also revealed further content needs and a desire for increased user input. Participants proposed adding functionalities for sharing reviews of digital health measurement instruments, suggesting website content, tracking progress within the stepped plan, and recording results per step.

**Figure 2:**
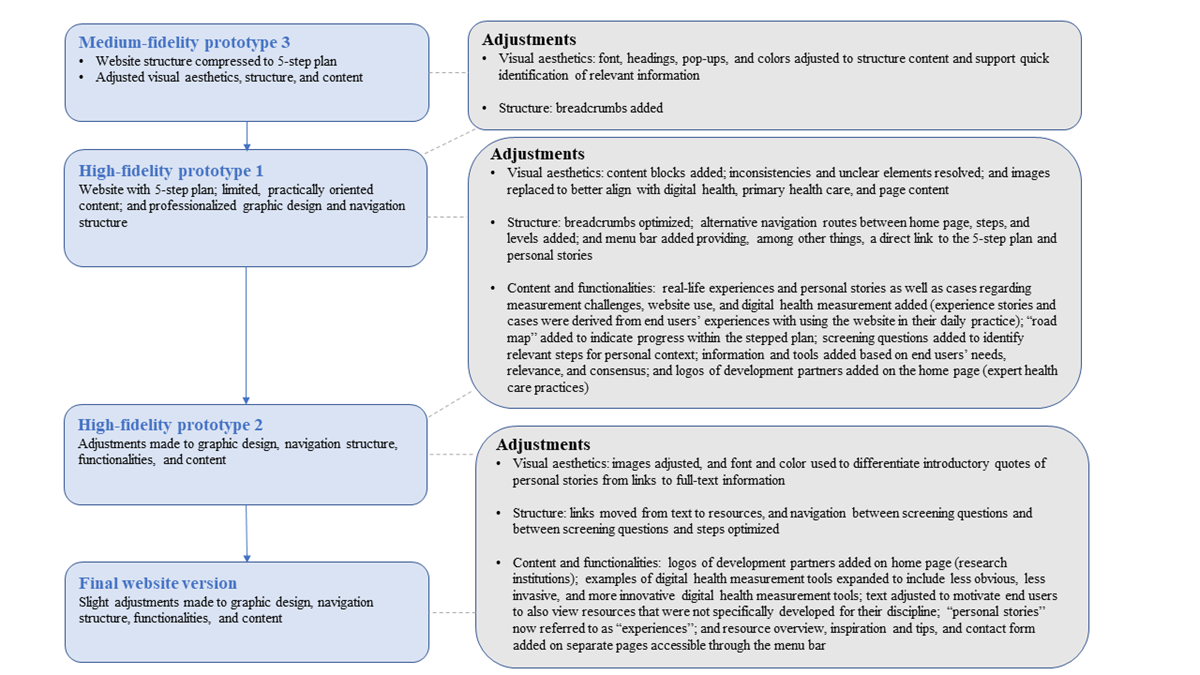
Adjustments made to iteratively advance medium-fidelity prototype 3 and the high-fidelity prototypes of the intervention designed to support primary health care professionals in digital health measurement into the final website version.

The findings from the first iteration were incorporated into high-fidelity prototype 2 (Figure 2). However, 3 adjustment proposals were deemed inapplicable or infeasible for this study. First, the researchers and website developers considered the website content too limited to justify a search function in addition to a menu bar. Instead, screening questions were introduced for identifying relevant content. Second, user-input was achieved by the aforementioned screening questions and by personal stories, instead of functionalities for directly sharing reviews and content. Third and last, recording individual results per step was excluded to avoid log-in requirements, maintaining accessibility and privacy.

#### Final Experiences and Adjustment Proposals

During the final iteration, 11 (100%) of the 11 participants to the usability testing completed the SUS for high-fidelity prototype 2, yielding a mean rating of 78.6 (SD 8.8), which indicates a “good to excellent” usability rating (percentile range 80-84) [73,74]. Apart from some minor issues, end users found the prototype easy to navigate, with consistent graphic design that enhanced predictability. The 5-step plan was considered logical and effective to guide clinical decision-making for the implementation of digital health measurement. Most end users described the content as practical, complete, concise, and well balanced between text and visuals:

You do get taken by the hand and the steps are logical and especially if you have a slightly less concrete measurement challenge, then I do find that you can really be helped in what possibilities there are.

Physical therapist, quartet 1

Newly added functionalities, such as screening questions and personal stories, were valued for directing users to relevant steps; enhancing recognition, motivation, and inspiration; and adding some “lightness” to the website:

Actually, you just can’t go wrong if you use this [screening questions]. If you end up in a strange place now, I would find that very odd.

Physical therapist, quartet 2

It should be an enjoyable website. Having “light” things in between makes it less clinical. It’s pleasant.

Speech and language therapist, quartet 2

If you land on a website, and you are looking for something, then, indeed, to read someone else’s stories, that gives you something to hold on to, and yes, maybe it feels more familiar, I don’t know, but I think it’s a good thing.

Occupational therapist, quartet 3

That’s something that a colleague may have experienced as well, because, we are people from practice...if you see that other people have tried something and they are enthusiastic about it.

General practitioner practice assistant, quartet 1

While the intervention’s generic approach was considered beneficial for interprofessional use, some end users suggested stronger recognition of specific health care disciplines through images or explicit mentions:

I would appreciate it if there would be a physical therapist or, you know, more of my profession in it.

Physical therapist, quartet 2

Other adjustment proposals concerned adding a separate resource overview and including a blog or forum to share reviews more actively.

The final website version included the requested resource overview. Images were fine-tuned to increase recognition for specific health care disciplines while preserving broad applicability. To avoid content monitoring challenges, the proposed blog or forum was replaced with a page featuring broadly scoped inspiration and tips from end users and a contact form for inquiries and suggestions. End users agreed with these changes.

**Figure 3:**
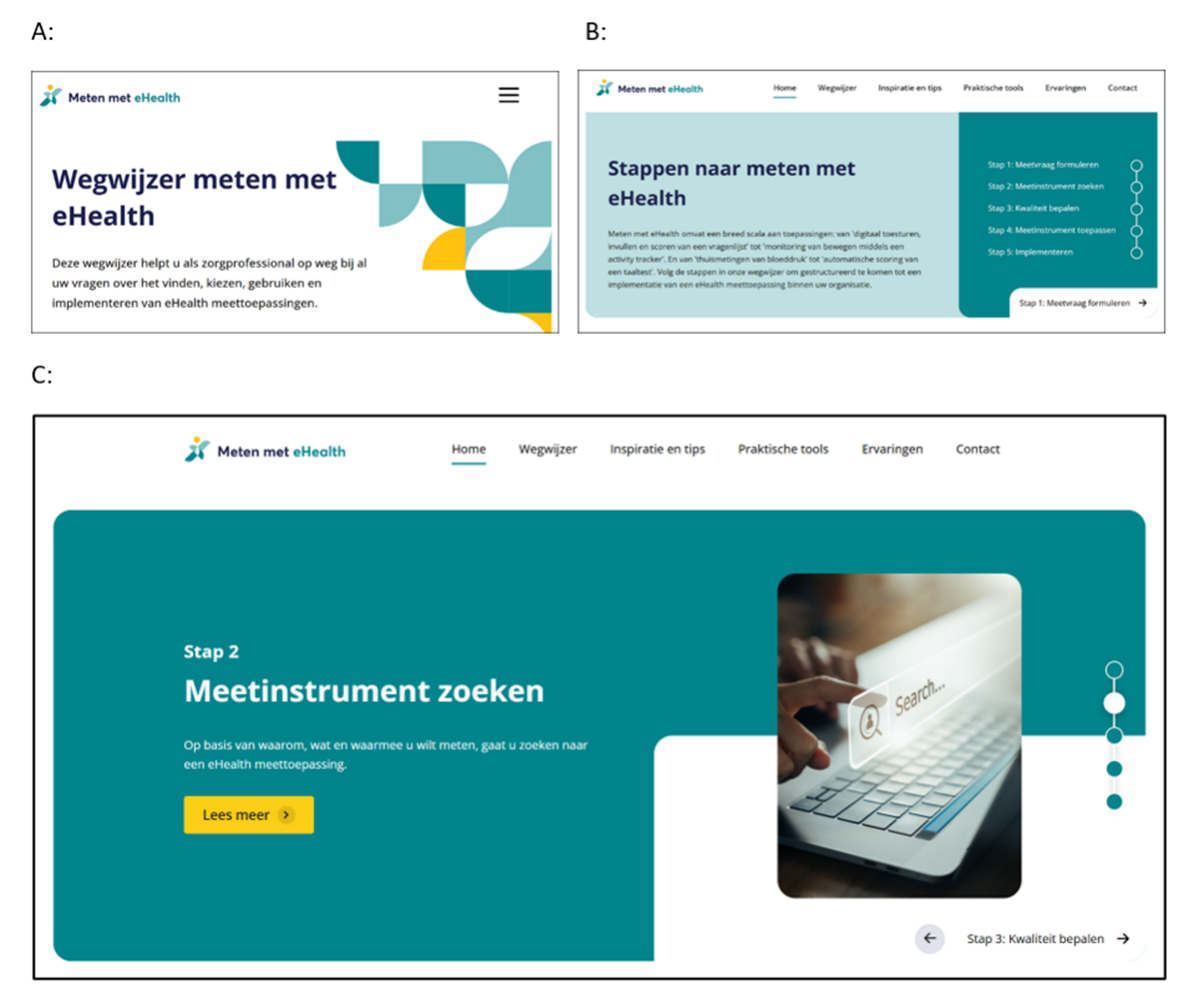
Screenshots of the final website version designed to support primary health care professionals in digital health measurement. (A) Title, subtitle, and logo. (B) Five-step plan for digital health measurement. (C) Basic content layer for step 2.

Figure 2 shows all major and minor adjustments integrated into the final website version, which was published in December 2023 [75] and is depicted in Figure 3.

## Discussion

### Principal Findings

This study used a PAR approach to develop an intervention to support sustainable, goal-oriented digital health measurement in primary practice while also stimulating change within participating practices. PAR prioritizes the integration of lived experiences and practical expertise as key knowledge sources [31,33]. In alignment with this approach, the study foregrounded end users’ experiences in identifying barriers, facilitators, and preferred strategies and resources for digital health implementation. Furthermore, researchers’ expertise and input from external experts were incorporated to enrich and triangulate these insights with theoretical frameworks.

Major barriers to digital health implementation identified in this study included limited knowledge and skills, time constraints within primary practice, and negative attitudes of health care professionals and patients. Facilitators included compatibility with team skills and external policies, positive attitudes of innovation enthusiasts, and the perceived advantages of digital health measurement for health care professionals and patients. These barriers and facilitators reflect the capability, opportunity, and motivation required for digital health performance, as outlined in the COM-B model, a scientifically validated behavior change framework that posits that for any behavior (B) to occur, a person must have the capability (C), opportunity (O), and motivation (M) to perform it [29,76,77]. Furthermore, they are consistent with barriers and facilitators reported for digital health implementation in general [6,20,25,26]. However, prior studies have also identified additional factors, including the influence of decision-making processes, improved patient-provider communication, and patients’ access to the internet [6,20,25,78]. These differences may be attributed to this study’s specific focus on barriers and facilitators experienced by health care professionals when implementing digital health for measurement purposes in Dutch primary care.

End users expressed support needs throughout the entire clinical decision-making process outlined in the “10-step plan for integrated use of measurement instruments in practice” [14,15]. However, to ensure the intervention’s feasibility, these 10 steps were consolidated into 5 overarching steps with concise and practical content. Engaging health care professionals in digital health implementation and enabling them to break the implementation process into smaller, manageable steps are shown to be promising strategies to improve capability and motivation for digital health performance [29]. End users identified a web-based, open-access format as the most suitable for delivering the intervention content. To meet their needs, graphic design, navigation and functionalities needed to be designed to ensure flexibility, personalized use, and quick identification of relevant information. This preference aligns with existing research showing that health care professionals favor information resources that are efficient, easy to use, and accessible without requiring a password or account, provided that the information is accurate [79,80]. To enhance trust in the accuracy of the website content, the website prominently displayed the practices and experts involved in its development—a strategy that may also improve digital health competency [29].

The intervention combined the website, which featured theoretical information, practical aids, examples, personal stories, and experiences, with coaching on the job. Together, these intervention components encompassed multiple strategies—education, persuasion, skills training, modeling, enablement, and empathic support—that are recognized as appropriate for enhancing capability, opportunity, and motivation for digital health performance [29,76,77]. However, while there is support for the effectiveness of both the website and coaching on the job stimulating digital health performance [29,76], in this study focusing on website development, coaching served as a temporary implementation strategy for the website and facilitated data collection for prototype development. Given its time-consuming nature, coaching may pose greater challenges for dissemination compared to the website and may need to be replaced by alternative implementation strategies that require less time.

The development process combined PAR and UCD principles to ensure that the intervention met the needs and routines of primary health care professionals. End-user involvement and iterative processes were essential throughout the reported UCD stages to enable the development of a user-friendly and suitable intervention. At the concept stage, comprehensive content needs were identified, aligning in part with core competencies and skills reported in previous research [81]. During the design stage, the necessity to focus on key content became evident, while the testing and trials stage highlighted the content that was essential. The emergent nature of the research design also proved invaluable. It allowed the PAR team to adapt to end users’ responses, such as addressing conceptual consensus issues by crafting a professional journey and strategically integrating disciplines into the team to facilitate the creation of early web-based prototypes.

### Strengths and Limitations

This study has several strengths. The study design facilitated flexible methods, active involvement of relevant stakeholders at all stages, and direct impact in participating practices [31,33-36,39,40,58,59]. At the same time, the fluid design posed challenges for maintaining consistency across data collection, analysis, and interpretation with respect to the research objectives and priorities. To ensure qualitative rigor, the study implemented triangulation, prolonged engagement, member checking, peer debriefing, and reflection, all of which are notable strengths. The intervention’s broad applicability across health care disciplines, digital health measurement technologies, and the entire clinical decision-making process is unique and aligns with evolving professional competencies and future health care developments [8,82,83]. To enhance transferability of the intervention within the Dutch primary health care context—particularly to disciplines that regularly conduct patient measurements to guide treatment, such as midwifery care and community nursing—the study intentionally included practices from 4 distinct health care disciplines, emphasized the interprofessional aspects of clinical decision-making, and used a generic framework [14,15]. To support scalability from the regional to the national level, the study incorporated a broad range of practice settings and engaged key national professional associations affiliated with the participating disciplines.

A limitation of this study was its reliance on photographs, forms, logs, reviewed documents, and minutes—rather than verbatim transcripts—for data collection and analysis during cocreative sessions in the concept and design stages [31,84,85]. This approach was adopted to encourage open expression and support a collaborative, nonhierarchical atmosphere between researchers and other participants. To enhance credibility, the minutes, interpretations, and conclusions underwent member checking through document reviews, real-time summaries, oral presentations, and iterative prototype feedback. Moreover, during the testing and trials stage, verbatim transcripts and quantitative questionnaires were used, contributing to extensive method triangulation. In addition, the sample did not include the “analog idealist” persona [52] because end users were recruited based on their willingness to enhance digital health measurement in daily practice. Although this limits the intervention’s applicability for this group, it aligns with the intervention’s objective of supporting primary health care professionals who are open to using digital health measurement. Persuading more reluctant health care professionals might require alternative strategies, such as incentivization [76,77], which were beyond the study’s scope. Another limitation was the underrepresentation of GP practice assistants compared to other primary health care disciplines. This was partially offset by the significant involvement of nursing lecturers and researchers among the external experts.

### Future Work

Although future research remains essential to guide broader adoption and scaling, the effectiveness of the intervention in fostering sustainable and goal-oriented digital health measurement by primary health care professionals—as well as the contribution of both the website and coaching on the job during this phase of intervention development—is already under study [68]. As successful intervention use in daily practice is influenced by both implementation processes and contextual factors [86], the ongoing combination of process and outcome evaluations is expected to yield meaningful insights into these dynamics and their impact on the use of goal-oriented digital health measurement. Moreover, as coaching may be difficult to disseminate due to constraints in time and personnel, the evaluation also examines its mechanisms of impact to inform the development of more scalable implementation strategies. The 4 included health care disciplines were selected to reflect a broader group of primary health care professionals with similar roles and responsibilities in measurement-based care. Future studies could explore whether adaptations are needed for specific professional groups. In addition, several practical needs and suggestions raised by end users were beyond this study’s scope and merit further investigation. These include developing an interdisciplinary database of digital health measurement instruments, facilitating the interprofessional use of digital health measurement, and exploring the intervention’s relevance for pre- and postlicensure education as well as secondary care.

### Conclusions

This study provides detailed insights into primary health care professionals’ format and content needs for an intervention to overcome barriers and leverage the potential benefits of sustainable and goal-oriented digital health measurement. Stepwise, easily accessible, visually appealing, and concise delivery of practical content—spanning the entire clinical decision-making process—proved crucial to facilitating the intervention’s integration into daily practice.

The development process described offers a structured approach to creating support strategies for digital health measurement in other contexts. Its PAR approach and UCD principles effectively engaged primary health care professionals in the cocreation of these strategies. Comparing the study’s findings with existing research and theoretical perspectives highlights significant alignment. This reinforces the relevance of these theories in practice while underscoring the value of end-user participation to tailor interventions to specific contexts.

Moreover, the study demonstrates that different health care disciplines and technology user personas can benefit from similar support strategies. Achieving this broad applicability requires a careful balancing of design and content.

## Appendix

Multimedia Appendix 1 Final version of a professional journey that linked intervention use to clinical practice.

Multimedia Appendix 2 The 10 usability heuristics formulated by Nielsen [66] and the 6 persuasive heuristics formulated by Cialdini [67] used to assess high-fidelity prototype 1.

Multimedia Appendix 3 Prototype screenshots: medium-fidelity prototypes 1 and 2.

Multimedia Appendix 4 Prototype screenshots: medium-fidelity prototype 3.

Multimedia Appendix 5 Website screenshots: final version.

Multimedia Appendix 6 Low-fidelity prototype 1 (columns 1 and 3), supplemented by MoSCoW (must have, should have, could have, would have) prioritization ratings (column 2).

## Abbreviations

COM-B: capability, opportunity, and motivation for any behavior to occur
GP: general practitioner
MoSCoW: must have, should have, could have, would have
PAR: participatory action research
SES: socioeconomic status
SRQR: Standards for Reporting Qualitative Research
SUS: System Usability Scale
UCD: user-centered design

## Data Availability

The datasets generated and analyzed during this study are available from the corresponding author on reasonable request.
