# Supplementary material for: Development of a Web-Based Intervention to Support Primary Health Care Professionals in Digital Health Measurement: User-Centered Participatory Approach
Source: JMIR Form Res. 2025 Sep 16;9:e72331. doi: 10.2196/72331 (PMC12485259; doi:10.2196/72331)
Supplement: Multimedia Appendix 1 [file formative_v9i1e72331_app1.pdf]

Multimedia Appendix 1: Final version of a professional journey that linked intervention use to clinical practice

Starting point 1: An individual client has an appointment with the healthcare professional in primary practice. Based on the client's care needs, the healthcare professional identifies measurement goals. **Starting point 2:** A (practice of) primary healthcare professional(s) wants to use digital health measurement more effectively and/or efficiently for specific target group(s). Both starting points lead to the following (thinking) steps:

Using clinical decision-making healthcare professional (together with client) establishes measurement goal(s) and measurement question(s).	Healthcare professional considers client and context and decides (together with client) to explore digital health measurement tools.	To gain insight into digital health measurement tools, healthcare professional can view various listing sites.	Depending on listing site, healthcare professional uses certain search filters and identifies potentially appropriate digital health measurement tool(s).
**#why #what**	**#which type**	**#find**	

Healthcare professional seeks information on reliability, validity and responsiveness of digital health measurement tool(s).	Healthcare professional reviews information on objective feasibility, such as administration procedure, ICT requirements, time and cost.	Healthcare professional also reviews information on subjective feasibility (from client's and professional's perspective), including ease of use, ICT skills and perceived usefulness.	Healthcare professional chooses (together with client) digital health measurement tool based on information gathered from previous steps, taking measurement goal(s), measurement question(s), client(s) and context as a starting point.
**#clinimetric_properties**	**#feasibility**		**#choose**

Healthcare professional informs client about use of chosen digital health measurement tool.<br><br>In case of active role for client: Healthcare professional gives instructions, taking into account client's health and digital skills.	Healthcare professional performs measurement (together with client).<br><br>In case of active role for client: Client performs measurement(s).	Healthcare professional analyzes and interprets (interim) relevant measurement results to ensure that they become meaningful and can be used in care and treatment process.	Healthcare professional uses measurement results to (together with client) clarify care needs, make diagnosis or prognosis, decide on treatment(s) or monitor and evaluate care. Healthcare professional can also combine data from multiple clients to gain insight to optimize the care process.
**#apply_and_interpret**			**#use_results**

In addition to (jointly) setting goals and choosing digital health measurement tools, healthcare professional takes his/her own attitude and ICT skills into account, and those of colleagues and clients.	Healthcare professional considers preconditions for digital health measurement within the healthcare practice, e.g. alignment with mission/vision and care/ICT processes, support from management/project leader, expertise/training and time/space for piloting.	Healthcare professional is aware of external developments regarding digital health measurement tools, such as inclusion within protocols and agreements with health insurers, and use by similar healthcare practices.	Sustainable implementation of digital health measurement requires preparation by healthcare professional, more specifically a planned approach, with relevant stakeholders, starting small and evaluating and adjusting regularly.
**#implement**			

This is a Multimedia Appendix to a full manuscript published in the J Med Internet Res. For full copyright and citation information see http://dx.doi.org/10.2196/72331.
