# Supplementary material for: Development of a Web-Based Intervention to Support Primary Health Care Professionals in Digital Health Measurement: User-Centered Participatory Approach
Source: JMIR Form Res. 2025 Sep 16;9:e72331. doi: 10.2196/72331 (PMC12485259; doi:10.2196/72331)
Supplement: Multimedia Appendix 2 [file formative_v9i1e72331_app2.pdf]

Multimedia Appendix 2: The 10 usability heuristics formulated by Nielsen and the 6 persuasive heuristics formulated by Cialdini used to assess high-fidelity prototype 1

Nielsen's ten usability heuristics [1]

1 Visibility of System Status:
The system should always keep users informed about what is happening through appropriate feedback within a reasonable timeframe.

2 Match Between System and the Real World:
The system should use language, concepts, and conventions that are familiar to the user, following real-world logic and avoiding technical jargon.

3 User Control and Freedom:
Users should be able to easily undo and redo actions, recover from errors, and navigate back to previous states without difficulty.

4 Consistency and Standards:
The interface should maintain internal consistency and adhere to established conventions and design standards to prevent confusion.

5 Error Prevention:
The design should proactively prevent errors by providing users with clear instructions, constraints, and timely warnings.

6 Recognition Rather Than Recall:
The system should minimize the user's memory load by making objects, actions, and options visible and easily retrievable when needed.

7 Flexibility and Efficiency of Use:
The interface should accommodate both novice and experienced users by allowing shortcuts, accelerators, and other efficiency-enhancing tools.

8 Aesthetic and Minimalist Design:
Interfaces should be visually clean and uncluttered, avoiding irrelevant or unnecessary information that can distract from core tasks.

9 Help Users Recognize, Diagnose, and Recover from Errors:
Error messages should be expressed in plain language, clearly indicate the problem, and suggest constructive steps for resolution.

10 Help and Documentation:
Although ideally unnecessary, help and documentation should be easily accessible, comprehensive, and focused on supporting users in effectively operating the system.

Cialdini’s six persuasive heuristics [2]

1 Social Proof:
Individuals tend to follow the behavior of others in situations characterized by uncertainty. Displaying positive reviews, testimonials, or user counts can foster trust and enhance perceived credibility.

2 Scarcity:
The perception that a product or opportunity is limited or exclusive can increase users’ motivation to engage. Highlighting limited stock, time-sensitive offers, or “while supplies last” messages can create a sense of urgency that prompts action.

3 Authority:
People are inclined to comply with individuals or institutions perceived as authoritative. Displaying certifications, logos of reputable organizations, or expert endorsements can strengthen user trust and perceived reliability.

4 Consistency, Reciprocity and Commitment:
People generally strive for consistency in their actions. Requesting small initial commitments and subsequently building upon them can enhance user engagement and increase the likelihood of further action.

5 Liking:
Users are more likely to respond positively to individuals or brands with whom they feel a connection. Demonstrating empathy, using visually appealing imagery, or cultivating a relatable and positive brand personality can foster user affinity.

6 Consensus (or Unity):
People often look to others’ behavior to determine what is normal or desirable. Displaying popularity metrics, social sharing buttons, or emphasizing widespread adoption can increase the perceived legitimacy and acceptance of a product or message.

[1] Nielsen J. Nielsen Norman Group. 2024 Jan 30. 10 usability heuristics for user interface design [accessed 2025-02-02] *https://www.nngroup.com/articles/ten-usability-heuristics/*
[2] Schenker M. CXL. 2024 Jul 30. How to use Cialdini’s 7 principles of persuasion to boost conversions [accessed 2025-02-02] *https://cxl.com/blog/cialdinis-principles-persuasion/*

This is a Multimedia Appendix to a full manuscript published in the J Med Internet Res. For full copyright and citation information see http://dx.doi.org/10.2196/72331.
