# Supplementary figures and images for: Development of a Web-Based Intervention to Support Primary Health Care Professionals in Digital Health Measurement: User-Centered Participatory Approach
Source: JMIR Form Res. 2025 Sep 16;9:e72331. doi: 10.2196/72331 (PMC12485259; doi:10.2196/72331)

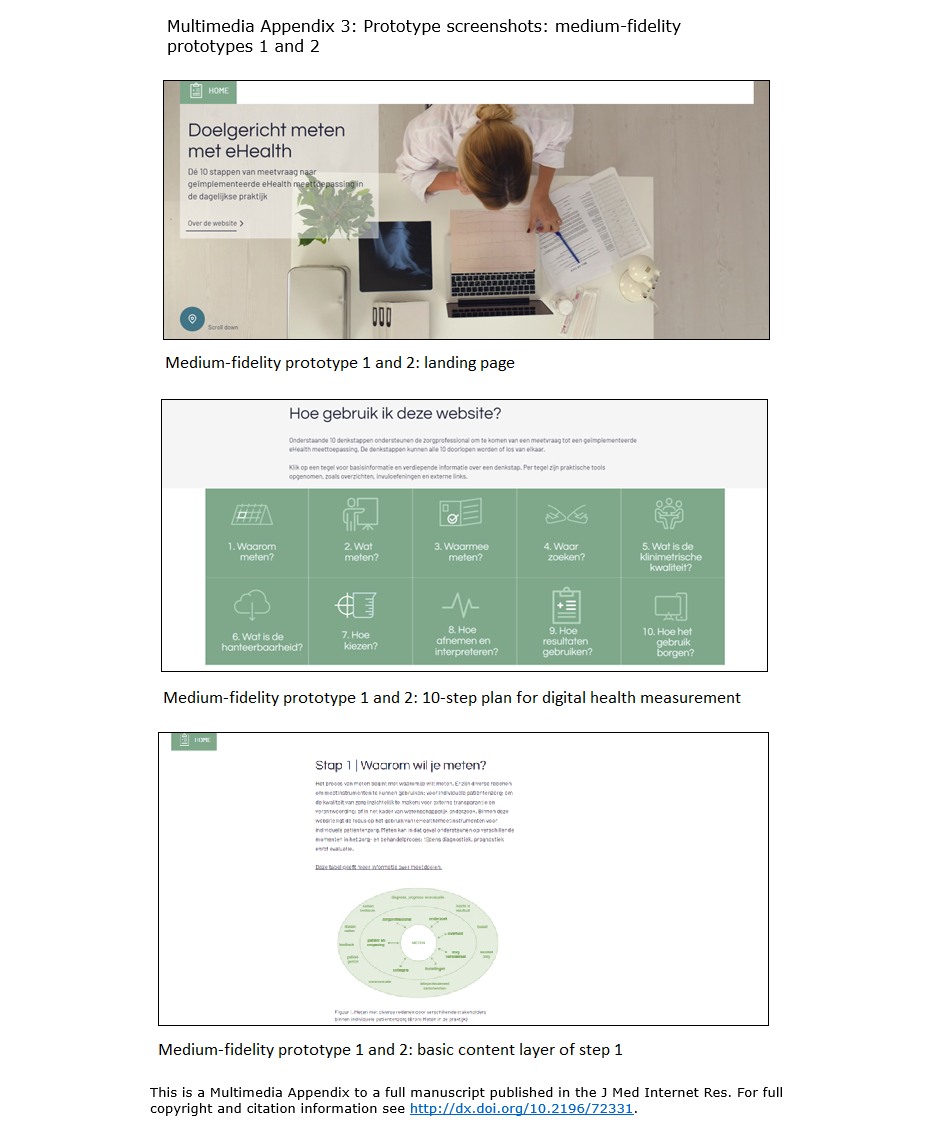

Supplement: Multimedia Appendix 3 [file formative_v9i1e72331_app3.png]

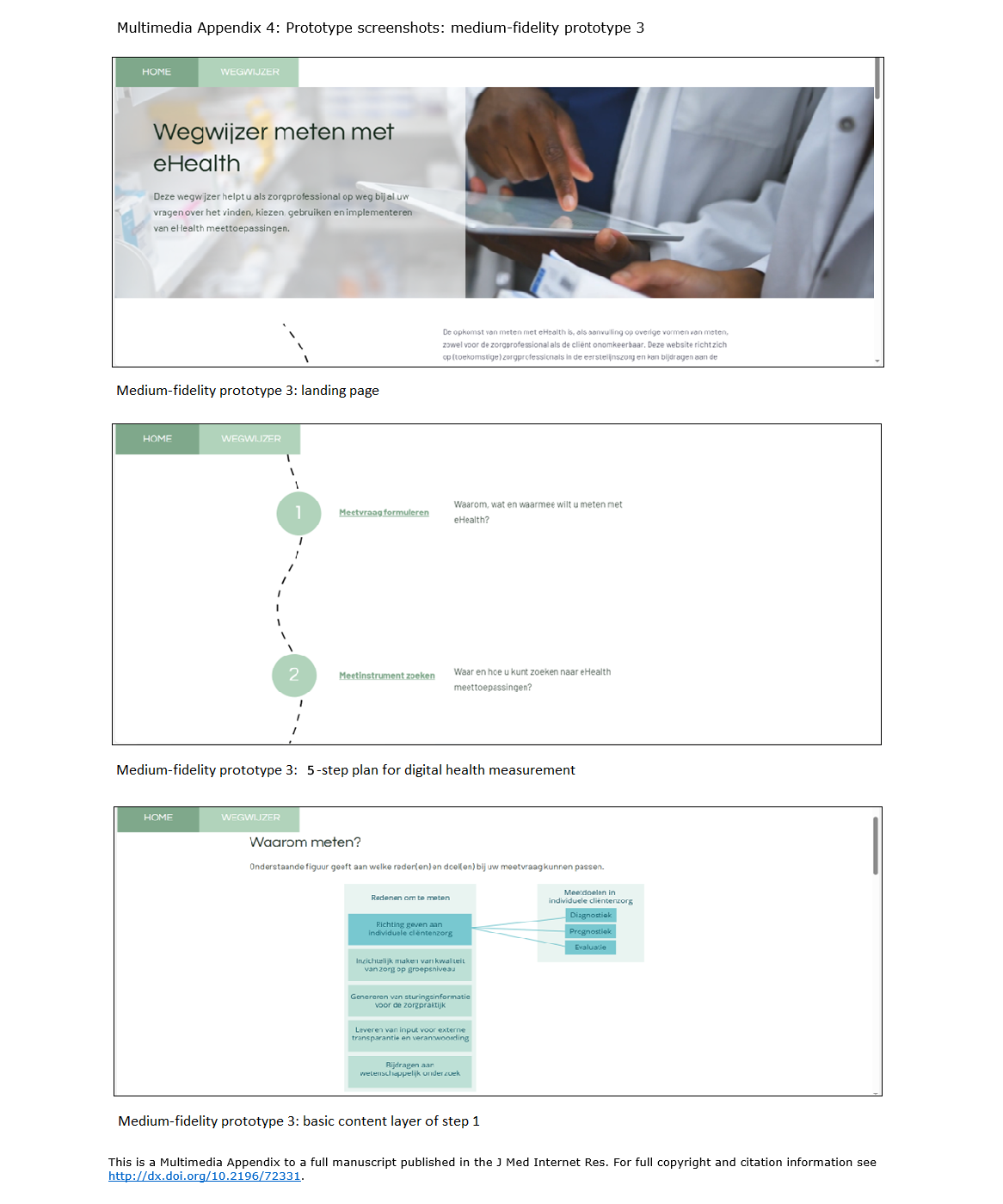

Supplement: Multimedia Appendix 4 [file formative_v9i1e72331_app4.png]

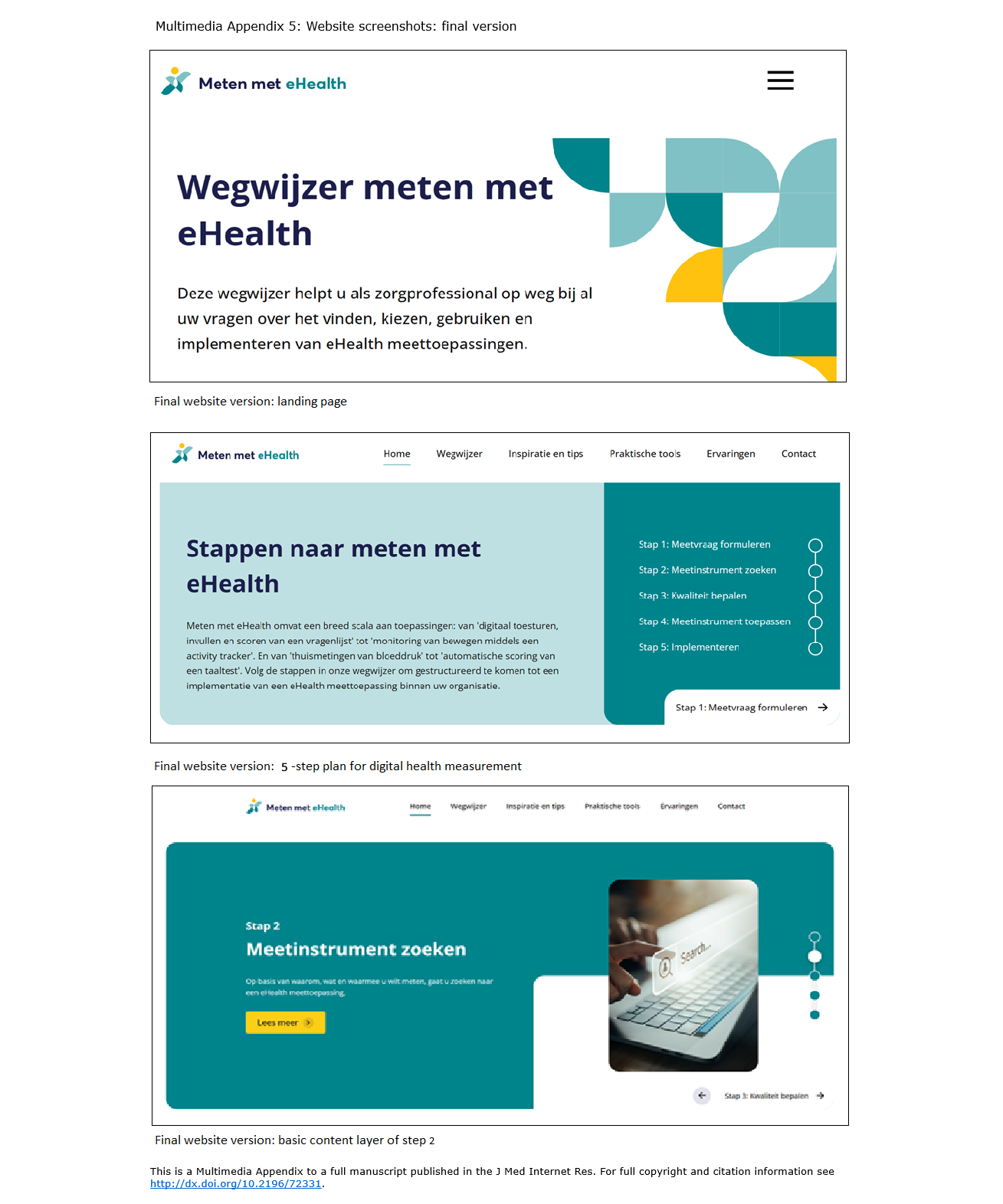

Supplement: Multimedia Appendix 5 [file formative_v9i1e72331_app5.png]
