# Supplementary material for: Development of a Web-Based Intervention to Support Primary Health Care Professionals in Digital Health Measurement: User-Centered Participatory Approach
Source: JMIR Form Res. 2025 Sep 16;9:e72331. doi: 10.2196/72331 (PMC12485259; doi:10.2196/72331)
Supplement: Multimedia Appendix 6 [file formative_v9i1e72331_app6.pdf]

Multimedia Appendix 6: Low-fidelity prototype 1 (columns 1 and 3), supplemented by MoSCoW (must have, should have, could have, would have) prioritization ratings (column 2)

10-step plan for integrated use of measurement instruments in practice [14,15], and support questions	MoSCoW ratings [1, 2] (provided by end users and the advisory board[a])				Relevant content categories as identified by end users and researchers
	Must have	Should have	Could have	Would have	
**Step 1: why do you want to measure?**					
1.1. "How do I determine why I want to measure (utilizing digital health)?"	2[b]	2[b]	N/A[c]	3[d]	• Theoretical information[e]<br>• Links to literature databases, toolkits, protocols, or guidelines[e]<br>• Practical tips for literature searching[e]
**Step 2: what would you like to measure?**					
2.1. "How do I determine what I want to measure (utilizing digital health)?"	4[b]	N/A	N/A	3[d]	• Theoretical information[e] and taxonomies[f]<br>• Relevant references[e]<br>• Practical resources: fillable forms for specifying measurement domains[f]

**Step 3: which type of measurement instrument is appropriate?**						
	3.1. “How do I determine what I want to measure with (type of measurement instrument, digital health yes or no)?”	3[b] and 4[d]	N/A	1[b]	N/A	• Theoretical information and taxonomies[f]<br>• Advantages and disadvantages of digital health measurement[f]<br>• Examples of measurement instruments and their applications[e]<br>• Practical resources: checklist and decision support tool on patients’ digital skills and blended therapy[f]
	3.2. “What forms of remote measurement can I use?”	2[d]	3[b]	1[b]	N/A	• Taxonomy and examples of digital health measurement instruments[f]
	3.3. “How do I determine whether to use digital health measurement?”[g]	N/A	N/A	N/A	N/A	N/A
**Step 4: how do you find a measurement instrument?**						
	4.1. “Where should I look for digital health measurement instruments?”	2[b] and 3[d]	N/A	2[b]	N/A	• Theoretical information[f]<br>• Links to websites[e] as well as guidelines and protocols[f] listing digital health

						(measurement) instruments[e]
	4.2. “How do I search for digital health measurement instruments?”	1[b] and 1[d]	N/A	1[b]	2[b]	• Practical tips on search strategies[f]
	4.3. “Does the patient already have access to a digital health measurement instrument?”[g]	N/A	N/A	N/A	N/A	N/A
**Step 5: what are the clinimetric properties?**						
	5.1. “Which clinimetric properties are important when assessing digital health measurement instruments?”	2[b]	2[b]	N/A	1[d]	• Theoretical information[e]
	5.2. “How do I assess the clinimetric quality of digital health measurement instruments?”	2[b]	1[b]	1[b]	3[d]	• Theoretical information[e] and knowledge clips[f]<br>• Links to databases with independent clinimetric assessments[e] and practical tips on search strategies[f]<br>• Practical resources: framework of clinimetric properties

						and assessment criteria as well as links to companies and websites providing assessment support[f]
	5.3. "Where can I find adequate information about the clinimetric quality of digital health measurement instruments? And if this quality is unknown, what do I do next?"[h]	N/A	N/A	N/A	N/A	N/A
**Step 6: what is the feasibility?**						
	6.1. "How do I meet ICT [Information and Communication Technology] requirements and General Data Protection Regulations?"	2[b] and 4[d]	1[b]	1[b]	N/A	• Theoretical information[e]<br>• Links to authoritative websites[e]<br>• Practical resources (factsheet, step-by-step plan, and checklist)[f]
	6.2. "Which usability aspects are important when assessing digital health measurement instruments?"	3[b] and 1[d]	N/A	1[b]	N/A	• Theoretical and practical information[e]<br>• Links to literature databases[e]<br>• Practical resources: guidelines, checklists, and stepped plans on

						usability, blended therapy, (health) literacy, communication, and digital personas[f]
	6.3. “How do I finance the utilization of digital health measurement?”	3[b] and 1[d]	N/A	1[b]	N/A	• Listing of financing options[e]<br>• Links to authoritative websites[f]
	6.4. “What is the (cost) effectiveness of digital health measurement?”	2[b] and 1[d]	2[b]	N/A	N/A	• Relevant references[f]
	6.5. “With whom (at individual/condition level) will I use digital health?”	3[b] and 2[d]	1[b]	N/A	N/A	• Practical guidelines or decision support tool[e]
	6.6. “How do I approach clients for participation in digital health innovation pilots within my health care practice?”	N/A	2[b]	2[b]	N/A	• Step-by-step implementation plan with practical guidelines[f]
**Step 7: how do you choose a measurement instrument?**						
	7.1. “How do I choose the most suitable digital health measurement instrument?”	2[d]	2[b]	2[b]	N/A	• Theoretical information[f]<br>• Decision support tool[f]<br>• Shared decision-making support tools[f]

**Step 8: how do you apply and interpret the results?**						
	8.1. "How do I instruct clients in the use of complex digital health measurement instruments, taking client diversity into account?"	3[b] and 4[d]	N/A	1[b]	N/A	• Theoretical information[f]<br>• Step-by-step instruction plan[e]<br>• Practical resources: guidelines, checklists, stepped plans, decision support tools, and factsheets on patients' digital skills, (health) literacy, and communication[f]
**Step 9: how are you going to use the results?**						
	9.1. "How do I use measurement results (towards clients and health insurers)?"	1[b] and 3[d]	N/A	3[b]	N/A	• Theoretical information[e]<br>• Practical guidelines and examples[f]<br>• Links to relevant literature[f]
	9.2. "How do I display relevant measurement results?"[i]	2[d]	2[b]	1[b]	N/A	• Practical guidelines[f]<br>• Links to authoritative websites[f]
	9.3. "How do I receive and integrate relevant measurement results into the electronic patient file?"	3[d]	2[b]	2[b]	N/A	• Theoretical information[f]

**Step 10: how do you implement the use of measurement instruments in daily practice?**						
	10.1. "Which barriers and facilitators are important when embedding digital health measurement within the health care practice?"[i]	1[b] and 2[d]	N/A	2[b]	N/A	• Theoretical information[f]<br>• Practical resources: guidelines, checklists, stepped plans, and toolkits for cocreation and for identifying barriers, facilitators, and implementation strategies[f]
	10.2. "How do I get inspired to explore opportunities for the utilization of digital health measurement?"[j]	1[d]	N/A	3[b]	1[b] and 1[d]	• Practical guidelines[f]<br>• Oversight of relevant sources[f]
	10.3. "How do I stay informed about innovations and share knowledge about them (with professionals, clients and their relatives)?"[j]	1[d]	2[b] and 1[d]	2[b]	N/A	• Practical guidelines[f]<br>• Oversight of relevant sources[f]
	10.4. "How do I share customized solutions for client instruction regarding the utilization of a digital health measurement instrument?"	1[d]	2[b]	2[b]	N/A	• Step-by-step implementation plan with practical guidelines[f]

[a]In the analysis, the "should have" and "could have" scores of the advisory board were excluded, as only "must have" and "would have" ratings were structurally gathered at the advisory board meeting.

[b]End users'ratings.

[c]N/A: not applicable.

[d]Advisory board ratings.

[e]Content categories identified by end users.

[f]Content categories identified by researchers.

[g]These support questions are already embedded or implied in overarching questions in the corresponding steps.

[h]This support question was added by members of the advisory board during application of the MoSCoW method and therefore not included in the "must have," "should have," "could have," and "would have" classification by the end users and the advisory board. However, as there was consensus within the advisory board on adding this question, it was considered essential for the low-fidelity prototype.

[i]One end user MoSCoW rating is missing.

[j]On the basis of the results of iteration 2, it was decided to combine support questions 10.2 and 10.3 in the further development of prototypes.

[1] Stevens A, van Dijk A, Jamin G, Beurskens A, Jorissen R. LIME. 2021. Co-creation impact compass: working together to foster impact [accessed 2024-4-25] *https://www.limeconnect.nl/wp-content/uploads/2021/07/Co-creation-Impact-Compass.pdf*

[2] Bradbury K, Watts S, Arden-Close E, Yardley L, Lewith G. Developing digital interventions: a methodological guide. Evid Based Complement Alternat Med 2014; 2014:561320. PMID 24648848, DOI 10.1155/2014/561320.

This is a Multimedia Appendix to a full manuscript published in the J Med Internet Res. For full copyright and citation information see http://dx.doi.org/10.2196/72331.
